# Effect of the electrode array-retina gap distance on visual function in patients with the Argus II retinal prosthesis

**DOI:** 10.1186/s12886-020-01631-6

**Published:** 2020-09-17

**Authors:** Abhishek Naidu, Nimra Ghani, Mohammad Saad Yazdanie, Khurram Chaudhary

**Affiliations:** Renaissance School of Medicine, Stony Brook University, Stony Brook, NY 11794 USA

**Keywords:** Argus II, Argus II retinal prosthesis, Direction of motion, Electrode array-retina gap distance, Gap distance, Low vision, Optical coherence tomography (OCT), Retinitis Pigmentosa, Square localization, Visual function

## Abstract

**Background:**

Post-implantation visual outcomes in patients with the Argus II Retinal Prosthesis is dependent on a multitude of factors including the positioning of the electrode array on the retina. The purpose of this study is to determine whether the average electrode array-retina gap distance correlates with objective visual function outcomes and sensitivity detection thresholds in patients implanted with the Argus II Retinal Prosthesis.

**Methods:**

Five patients with implantation of the Argus II Retinal Prosthesis were enrolled in this single-institution retrospective study. Patient demographics were collected from medical records. Visual function data (Square Localization [SL] and Direction of Motion [DOM]) and Optical Coherence Tomography (Cirrus HD-OCT) images were extracted retrospectively from the Argus II Retinal Prosthesis Post-Approval study. Visual function tests were performed with the device OFF and ON at each study visit. Electrode array-retina gap distances were measured at each of the array’s 60 electrodes using the Cirrus HD-OCT software in both the nasotemporal and superoinferior planes. Data was obtained at baseline, and post-operative month 1, month 3, month 6, and year 1. Sensitivity detection thresholds were obtained at the initial programming visit and each reprogramming session.

**Results:**

Three patients performed significantly better in SL visual function testing with the device ON. Patients that worsened in visual function testing with the device ON in both SL and DOM testing had a statistically significant decrease in performance. The electrode array-retina gap distance was found to effect performance in SL testing in a patient-dependent manner. No effect was found between the electrode-array gap distance and DOM testing or sensitivity detection threshold.

**Conclusion:**

Our results demonstrate that the electrode array-retina gap distance may affect visual function outcomes in SL testing in certain patients with the Argus II Retinal Prosthesis, and the direction and magnitude of this effect is likely patient-dependent. Furthermore, complete apposition between the electrode array and retina may not always be necessary to achieve optimal visual outcomes.

## Background

Retinitis Pigmentosa (RP) is an inherited degenerative disorder resulting in the loss of photoreceptor cells of the outer retina. Over 1 million patients worldwide suffer from RP, which leads to progressive vision loss that can pose significant challenges in many facets of their lives. More than 190 genes have been implicated in the pathogenesis of RP and all mutations inevitably result in degeneration of the photoreceptor layer [1]. However, the inner retinal cells are largely preserved in RP, serving as a target for direct electrical stimulation in order to generate phosphenes that may be perceivable by the patient [2]. Producing and manipulating these phosphenes to accurately reflect the surrounding visual environment is the common objective for electric stimulation-based visual prostheses.

The Argus II Retinal Prosthesis System (Second Sight Medical Products Inc., Sylmar, California, USA) was developed for the purpose of providing artificial vision in patients with outer retinal degenerative diseases such as RP. It became commercially available after receiving FDA approval as a humanitarian device in 2013. Roughly, the Argus II consists of an implantable epiretinal 60-channel electrode array connected wirelessly to a portable computer (Visual Processing Unit, VPU). The VPU receives real-time images from a video camera mounted onto glasses worn by the patient. Wireless connection between the electrode array and the VPU occurs via an external coil (side arm of the glasses) and an internal coil (housed in the internal unit) that utilize radiofrequency telemetry. An application-specific-integrated-circuit (ASIC) within the internal unit generates electrical impulses in accordance with the VPU output. These impulses are relayed to the electrode array, ultimately stimulating the inner retina in order to produce an artificial image [1, 2].

Surgical techniques for implantation of the Argus II retinal prosthesis may vary depending on patient factors, globe anatomy, and surgeon preferences. Array positioning over the macular region is a requisite for optimal visual outcomes [3]. Normally, a spring-tensioned titanium retinal tack is used to affix the electrode at the posterior pole in order to ensure close apposition of the electrode to the retinal surface. However, even with the concavity of the electrode, complete apposition of the electrode array against the retina may not occur. Ahuja et al. [3] demonstrated that complete or near complete apposition results in a lower perceptual threshold. However, the effect of the electrode array-retina gap distance on objective visual function outcomes has not been elucidated. This study aims to determine how the electrode array-retina gap distance influences postoperative visual outcomes in patients with RP implanted with the Argus II retinal prosthesis, with the hypothesis that smaller gap distances correlate with more significant visual improvement.

## Methods

### Patient eligibility and demographics

Patients enrolled in the Argus II Post-Approval Study (PAS) were invited to join this study. Eligibility criteria included a diagnosis of outer retinal dystrophy, some residual light perception, ability to undergo surgery under anesthesia, and ability and willingness to comply with post-operative follow-up and testing. Exclusion criteria included co-morbid ocular conditions (e.g. advanced glaucoma, optic glioma) that could compromise the functional optic nerves and tracts. Pretesting at the baseline visit included visual function testing, general slit lamp examination, Optical Coherence Tomography imaging (Cirrus HD-OCT Machine, Carl Zeiss Meditec, Dublin, Calif.) and fundus photography. A total of 5 patients with implantation of the Argus II device were enrolled in the study (Table 1). All patient recruitment, study investigations and surgeries were conducted at Stony Brook University Hospital and its affiliated sites. Surgeries were performed by one vitreoretinal surgeon under general anesthesia at the Stony Brook University Hospital.

**Table 1:** Demographics of patients included in the Argus II Retinal Prosthesis study at baseline

Patient #	Sex	Age	Diagnosis	Years since Diagnosis	Visual Acuity at Presentation
**1**	F	60–70	Leber’s Congenital Amaurosis	Unknown	Bare Light Perception (BLP)
**2**	F	50–60	Retinitis Pigmentosa	19	Bare Light Perception (BLP)
**3**	M	70–80	Retinitis Pigmentosa	28	Bare Light Perception (BLP)
**4**	M	70–80	Retinitis Pigmentosa	48	Bare Light Perception (BLP)
**5**	M	60–70	Retinitis Pigmentosa	33	Light Perception (LP)

### Data collection

Data was collected from patient visits at baseline prior to implantation, post-operative month 1 (M1), month 3 (M3), month 6 (M6), and year 1 (Y1). At M1, activation and programming of the device took place and visual function testing was deferred. Visual function testing was performed at M3, M6 and Y1. Sensitivity detection thresholds (to be defined later) were collected when possible at M1, M3, M6 and Y1. Gap distances were measured from Optical Coherence Tomography (OCT) images at M1, M3, M6, and Y1. All data was collected by two authors, cross-checked for accuracy and thoroughness, and independently reviewed by two other authors. Microsoft Excel was utilized for data collection.

### Visual function

The visual function of patients with the Argus II Retinal Prosthesis was assessed using Square Localization (SL) and Direction of Motion (DOM) measures which were developed by Second Sight. SL involves locating and touching a white square set against a black background on a touchscreen monitor with the device ON and OFF. The distance (measured in number of pixels) between the true center of the square and the location at which the patient touches the monitor where they perceive the center of the square to be is measured, with smaller pixel distances indicating higher accuracy [2]. DOM testing utilizes a white bar that moves across a black screen in a particular direction, and the patient attempts to determine the direction of motion of the white bar. The angle between the true direction of motion and the patient’s perceived direction of motion is measured, with smaller angles indicating higher accuracy [2] All testing was performed under controlled conditions and overseen by trained personnel. Visual function data was extracted retrospectively from the Argus II Post-Approval Study.

### Sensitivity detection threshold

The Argus II device is programmed at M1 for initial programming prior to turning on the camera in order to find stimulation levels that are suitable for the patient to be set on their Video Processing Unit (VPU) [4, 5]. During the first session, the electrodes whose resistances are too high are disabled and the electrodes that are functional and usable are determined. A quick scan of the array is done using various stimulation amplitudes to determine which electrodes are able to produce a percept or phosphene. The minimum current that is needed for the patient to see a phosphene 50 % of the time is defined for each electrode [1, 4]. The video signal from the camera is mapped to the electrical signal for individual or groups of electrodes, determining which electrodes are being stimulated and at what respective frequency. These values are set to the patients VPU to be used in different conditions [1]. Programming for this study included use of the “Programming Assistant” software that is designed to streamline the programming process to measure general sensitivity [1, 4]. Through use of this software, the camera alignment process is simplified and comfort is determined under more real-world stimulation conditions [1]. Depending on the patient experience, the device can be reprogrammed on a regular basis for a readjustment in response to changes to the array or the patient’s responses to the electrode stimulation [5, 6]. The brightness of perception and the number of electrodes that give the patient a perception may decrease over time. Patients may need several reprogramming sessions in order to fully optimize the use of the device [5]. The sensitivity detection from each programming session for each patient was provided by from the programming technician from Second Sight where the values for sensitivity (μA) of each electrode for each session and the maximum current (μA) of each electrode per stimulation were collected and analyzed. Unfortunately, sensitivity detection thresholds were not collected for every patient at the scheduled sessions due to various logistical issues.

### Electrode array-retina apposition distance (gap distance)

**Fig. 1:**
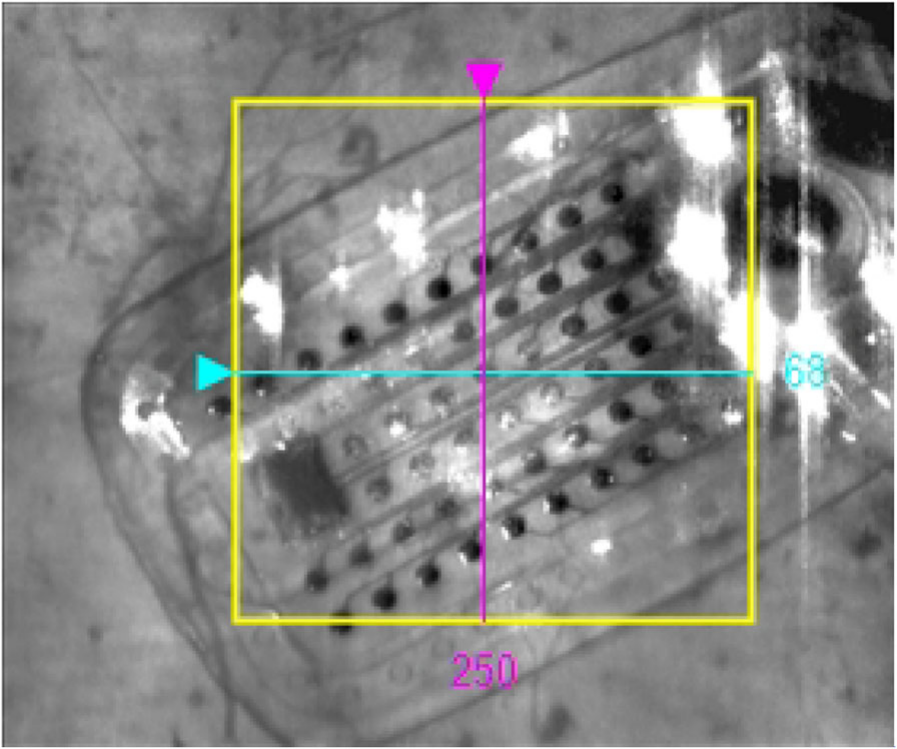
OCT macular cube fundus view depicting electrode array positioned over macula. The macular cube scan in the Zeiss OCT software was used to measure the electrode array-retina gap distance at each individual electrode. 60 electrodes per array in a 6 × 10 grid were labeled as A-F and 1–10. Perpendicular nasotemporal and superoinferior raster lines were positioned to intersect at the center of the desired electrode using the fundus view

**Fig. 2:**
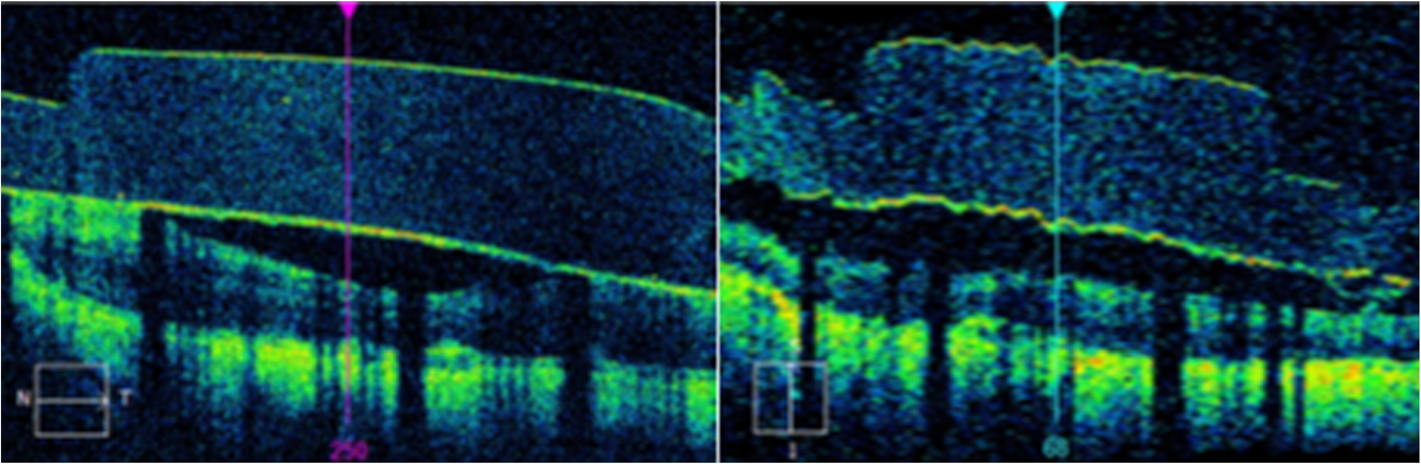
OCT macular cube horizontal and vertical tomograms. After targeting a specific electrode with the raster lines, the software’s caliper measurement tool was used to measure the distance along the raster lines between the electrode array and the surface of the retina on the associated horizontal and vertical tomograms

A Cirrus HD-OCT Machine was utilized by the PAS study to capture images during each patient visit. Parameters for capture at each visit included a 6 mm × 6 mm macular cube image (acquiring 512 nasotemporal scans × 128 superoinferior scans) which were utilized for study purposes. Images were located using unique patient identifiers and analyzed using Zeiss OCT analytical software. Eligible scans included a majority of the 60 electrodes and a surrounding portion of retina, the ability to visualize or estimate the precise location of each electrode, and a signal strength of at least 5 out of 10 (determined by the Zeiss software). While in the macular cube view of the software, the perpendicular nasotemporal and superoinferior raster lines were placed directly over the center of each visualized electrodes (Fig. 1). On the associated horizontal and vertical tomograms, the built-in caliper measurement tool was used to measure the vertical distance (in μm) between the electrode array and retina (Fig. 2). These measurements were taken for each patient at M3, M6 and Y1. The electrodes were designated a label (A-F, 1–10) to ensure consistency of measurements at each visit. Out of 1200 electrodes, 1124 (93.7%) were able to be measured.

### Statistical analysis

At each visit for SL and DOM testing, results were documented with the device OFF and ON. The difference in pixel distance (SL testing) or degrees (DOM testing) measured as the testing performance with the device OFF minus ON (Δ^OFF-ON^) was used to determine visual function changes with the Argus II Retinal Prosthesis. A positive Δ^OFF-ON^ indicated an improvement in functional visual testing with the device ON. The unit of analysis was the patient with the assumption that visual function performance was patient-dependent. As such, Wilcoxon Signed-Rank tests were used with paired samples for each individual patient as well as for patients that improved or worsened with the device ON to determine whether Δ^OFF-ON^ was significant. Non-parametric testing was chosen due to the small sample size and because no assumptions were made about the normality of the distribution in testing performance. To correlate visual function with gap distance, generalized linear models (GLMs) using Δ^OFF-ON^ for SL and DOM testing (dependent variables), and gap distances (covariates) were used. A separate model examining the interaction between patient and gap distance as predictor variables was performed to determine the patient-dependent effects of gap distance on visual function performance. GLMs were also used to determine the effect of sensitivity detection threshold on average Δ^OFF-ON^. Statistical analyses were performed using IBM SPSS Version 27 (IBM Corp., Armonk, N.Y., USA).

## Results

### Correlation of electrode-retina gap distance with sensitivity detection threshold

The sensitivity detection threshold measured for a patient at M1, M3, M6 or Y1 corresponded to the average electrode array-retina gap distance at the same visit, as the OCT image was captured on the same day. Using a GLM with pooled data, gap distances were found to have no significant effect on sensitivity detection thresholds (*p* > 0.05). A separate interaction model found no significant effect of gap distance on sensitivity detection threshold when taking patient-dependent effects into account (*p* > 0.05) (Table 2).

**Table 2:** Results of sensitivity detection threshold testing at month 1 (M1), month 3 (M3), month 6 (M6), and year 1 (Y1) measured as the minimum current needed for a patient to see a phosphene 50% of the time

Sensitivity (μA)					
Patient #	M1	M3	M6	Year 1	Average
**1**	120.41	–	–	–	**120.41**
**2**	66.13	136.8	156.2	–	**119.71**
**3**	141.05	–	–	–	**141.05**
**4**	143.35	–	–	–	**143.35**
**5**	130.96	190.73	218.62	197.89	**184.55**

### Visual function outcomes

**Fig. 3:**
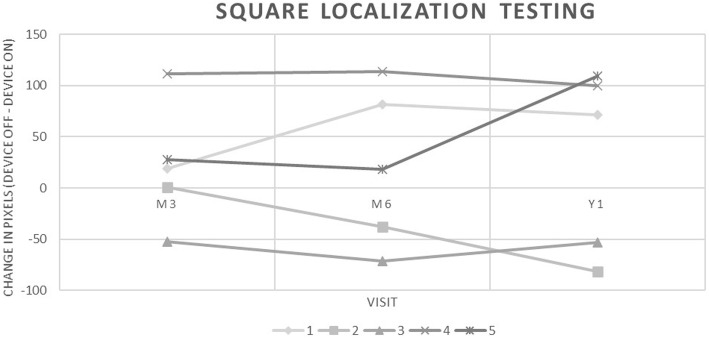
Square Localization Testing results for study patients at month 3, month 6, and year 1 measured as a change in pixels between testing with the device OFF and ON. A positive number indicates an improvement with the device ON

**Fig. 4:**
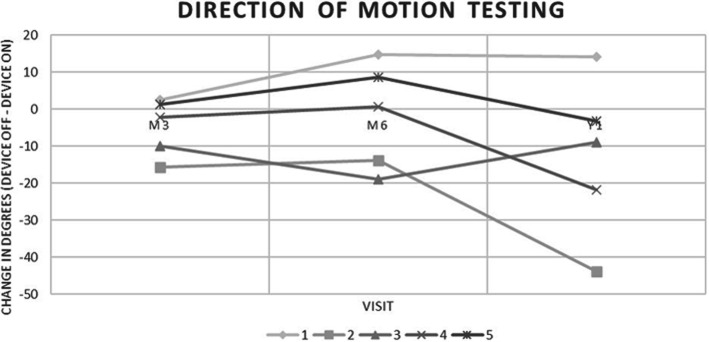
Direction of Motion Testing results for study patients at month 3, month 6, and year 1 measured as a change in degrees between testing with the device OFF and ON. A positive number indicates an improvement with the device ON

Using Wilcoxon Signed-Rank tests to compare testing performance with the device OFF and ON for each patient individually, no statistically significant difference was found for any patient in both SL and DOM testing. We then analyzed testing performance for patients that had an average positive Δ^OFF-ON^, average negative Δ^OFF-ON^, and with pooled data to determine if these differences were significant. In SL testing, 3 out of 5 patients demonstrated an improvement in visual function at M3, M6 and Y1 with the device ON (Table 3, Fig. 3), indicated by an average positive Δ^OFF-ON^. These improvements were found to be statistically significant (*p* < 0.05). In patients with an average negative Δ^OFF-ON^, this difference was also found to be statistically significant (*p* < 0.05). Using pooled data from all patients, Δ^OFF-ON^ for SL testing was not statistically significant (*p* > 0.05). In DOM testing, 2 patients demonstrated a positive Δ^OFF-ON^ when averaged over the three visits, with 1 patient having an improvement at all testing visits (Table 3, Fig. 4). However, paired Wilcoxon Signed-Rank Tests for patients with an average positive Δ^OFF-ON^ and for a pooled sample with all patients were not statistically significant (*p* > 0.05). The difference in patients with a negative Δ^OFF-ON^ was found to be statistically significant (*p* < 0.05).

**Table 3:** Results of Square Localization (SL) and Direction of Motion (DOM) Testing at month 3 (M3), month 6 (M6), and year 1 (Y1) measured as a change in pixels (SL) or degrees (DOM) between testing with the device OFF and ON (Δ^OFF-ON^). A positive number indicates an improvement with the device ON

	Square Localization Testing				Direction of Motion Testing			
	Δ^**OFF-ON**^ (pixels)				Δ^**OFF-ON**^ (degrees)			
**Patient #**	**M3**	**M6**	**Y1**	**Average**	**M3**	**M6**	**Y1**	**Average**
**1**	18.67	81.47	71.14	**57.09**	2.39	14.74	14.20	**10.45**
**2**	0.90	− 37.71	− 81.36	**−39.39**	− 15.58	−13.87	−43.74	**−24.39**
**3**	− 52.20	−71.52	−52.72	**−58.81**	−10.03	−19.02	−8.94	**−12.66**
**4**	111.50	113.72	100.17	**108.46**	−2.10	0.63	−21.80	**−7.76**
**5**	27.96	18.35	108.99	**51.76**	1.32	8.54	−3.21	**2.22**

### Correlation of visual function with gap distance

**Fig. 5:**
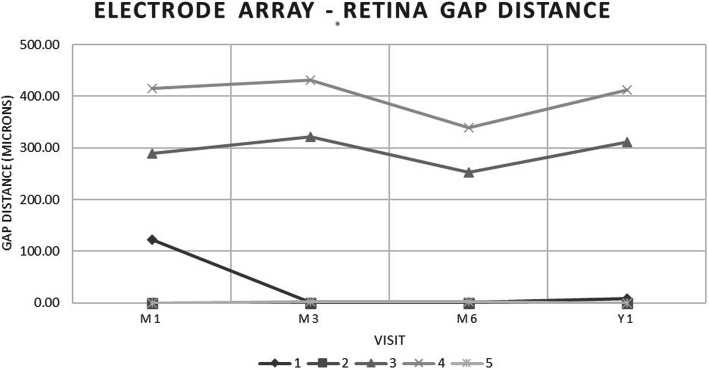
Post-implantation electrode array-retina gap distances of study patients averaged over all measured electrodes at month 1, month 3, month 6 and year 1

Using pooled data, no significant effect was found between Δ^OFF-ON^ in SL and DOM testing and electrode array-retina gap distance (Fig. 5). When accounting for patient-dependent effects using an interaction model, a significant effect was seen between electrode array-retina gap distances and Δ^OFF-ON^ in SL testing (*p* < 0.05). The β coefficients for patients 3 and 4 where the most statistically significant effects were seen were − 0.240 and 0.237, respectively, indicating that the directionality of a patient-dependent effect of gap distance of SL testing performance may be positive or negative. This patient-dependent effect was not seen between electrode array-retina gap distances and Δ^OFF-ON^ in DOM testing (*p* > 0.05).

## Discussion

The Argus II retinal prosthesis is a relatively novel medical device that can marginally restore some functional vision in patients suffering from retinal dystrophies. Numerous studies have demonstrated that a statistically significant improvement in visual function can occur in some patients, and it can no doubt impact their lives tremendously [7–10]. Our study’s analysis of SL results in patients with the device further demonstrates that a significant improvement in visual function is possible, as the three patients that performed better with the device ON in SL testing had a statistically significant improvement. Conversely, the patients that performed worse with the device ON in SL and DOM testing had a statistically significant decrease in performance. An exploration of the factors that influence these positive or negative outcomes is crucial to our understanding of how to further improve visual prosthetic technology. Our study aimed to determine how the gap distance correlates with functional measures of visual acuity such as Square Localization and Direction of Motion testing.

As our results demonstrate, electrode array-retina gap distance had a significant effect on SL testing performance in our patient cohort when considering patient-dependent factors. These factors may include the electrode, the globe, and patient behavior. Due to these patient-dependent factors, the gap distance alone could not predict their visual function outcomes in our study. We speculate that gap distance may influence visual function outcomes in certain patients, though the direction of the effect may be difficult to ascertain without formal testing. The patient with the largest average gap distance (patient 4) had the greatest average improvement in SL testing Conversely, the patient with complete apposition of the electrode (patient 2) at all visits demonstrated decreased visual function in both SL and DOM testing. Furthermore, patient 3 had a decrease in SL testing performance while patient 4 had an increase in SL testing performance when there was a decrease in their gap distances. We were unable to find a significant patient-dependent effect of gap distance on DOM testing. Based on our results, we reject our hypothesis that smaller gap distances correlate with more significant visual improvement. In future iterations of the device, post-operative manipulation of the electrode-array retina gap distance may help in optimizing a patient’s visual function outcomes.

While the effect of the electrode-array retina gap distance on visual function could not be predicted, we believe that complete apposition of the electrode array on the retina may not be necessary for optimal visual outcomes in all patients. This becomes a crucial element in determining patient eligibility for patients with anatomical defects that could affect apposition. Rizzo et al. [2] and Delyfer et al. [11] have demonstrated that complete apposition of the electrode array on the retina may not be possible in patients with a posterior staphyloma. However, our results demonstrate that patients with staphylomas may not necessarily be precluded from implantation of the Argus II retinal prosthesis, as visual improvement may still occur in the presence of an electrode array-retina gap. An improvement in visual function in patients with a posterior pole staphyloma implanted with the Argus II retinal prosthesis has been demonstrated by Seider et al. [12]. It is possible that other staphyloma factors such as size, shape, and location may affect visual function outcomes rather than its presence alone.

It has been previously determined that the electrode array-retina gap distance affects electrical threshold in patients implanted with the Argus II retinal prosthesis, with complete apposition correlating with lower perceptual thresholds [3]. Our study did not find an effect of gap distance on sensitivity detection thresholds. However, our analysis of this correlation was limited by numerous missing values, as the sensitivity detection threshold testing was not performed for most patients at all study visits. A larger sample size would be necessary to further study this correlation.

One patient with almost complete apposition of the electrode array on the retina (patient 1) developed clinically significant retinoschisis. It is possible that complete apposition of the array may increase the risk of adverse postoperative outcomes due to traction, overstimulation of the retina, and inflammatory changes resulting in remodeling of the inner retinal layers.

SL and DOM testing are inherently arduous and time consuming, requiring patients to remain focused and motivated in order to perform optimally. Testing results may be influenced by frustration and avolition, although all attempts were made to ensure that patients completed testing in a comfortable environment. One patient (patient 3) was on an SSRI antidepressant during all testing visits and seemed more motivated to perform well. SL and DOM testing although requires some degree of hand-eye coordination, a skill which may have slightly regressed in these patients due to a long-standing history of blindness. Testing methods that are less time consuming and easier for low-vision patients to perform would allow for a more accurate determination of post-operative visual function. Subjective measures that allow for a better assessment of what patient goals for the device may be and how they hope the device could be used to achieve them would also provide more crucial information for the device’s potential for patient benefit. Additionally, optimal utilization of the Argus II retinal prosthesis requires visual rehabilitation and self-learning by the patient during their day-to-day life. Depending on the quality and quantity of visual rehabilitation, and the amount of time a patient spends with the device turned ON, their ability to interpret the generated phosphenes would be affected. It is also possible that overuse of the device could negatively impact visual function through constant electrical stimulation. These factors could ultimately influence performance on SL and DOM testing. Lastly, we noted that gap distances changed over time, with three patients having changes of over 50 μm between testing visits. This may be due to the positioning tack coming into contact with extraocular muscles at the scleral interface, or anatomical changes post-operatively.

Our study is limited by a small sample size (*n* = 5), as our study data and patients were from a single site. We also believe that comparing visual function with the device OFF and ON using non-parametric testing for each patient individually may have failed to identify significant differences (type II error), as each patient had only 3 related measurements. Lastly, although we were unable to find an effect of the electrode array-retina gap distance on sensitivity detection thresholds or DOM testing, we cannot make conclusions about these correlations due to our limited data. Future studies with a larger data set may provide any opportunity to further explore the benefits of the Argus II Retinal Prosthesis and the relationship between the electrode-retina gap distance and visual outcomes.

## Conclusion

Our study demonstrates that the post-operative electrode array-retina gap distance may affect objective visual function outcomes for SL testing in certain patients implanted with the Argus II Retinal Prosthesis. Patient-dependent factors likely influence how the electrode functions in each patient. This finding is an important consideration in patient selection criteria and surgical methods. Complete apposition of the electrode array on the retina is likely not necessary in all patients for optimal post-operative visual outcomes. In fact, complete apposition could possibly lead to adverse effects due to traction placed on the retina or overstimulation of the inner retinal cell layers. Furthermore, determining how individual patients’ visual function changes with the electrode array-retina gap distance may help to optimize their visual outcomes.

## Data Availability

The datasets used and/or analyzed during the current study are available from the corresponding author on request.

## Abbreviations

SL: Square Localization
DOM: Direction of Motion
HD-OCT: High Definition-Optical Coherence Tomography
RP: Retinitis Pigmentosa
VPU: Video Processing Unit
OCT: Optical Coherence Tomography
M1: Month 1
M3: Month 3
M6: Month 6
Y1: Year 1
